# ^99m^Tc-labeled benzenesulfonamide derivative-entrapped gold citrate nanoparticles as an auspicious tumour targeting

**DOI:** 10.1038/s41598-025-88862-z

**Published:** 2025-02-08

**Authors:** Adli A. Selim, Tamer M. Sakr, Basma M. Essa, Galal H. Sayed, Kurls E. Anwer

**Affiliations:** 1 Labelled Compounds Department, Egyptian Atomic Energy Authority (EAEA), Cairo, 13759 Egypt; 2 Radioactive Isotopes and Generators Department, Egyptian Atomic Energy Authority (EAEA), Cairo, 13759 Egypt; 3 Heterocyclic Synthesis Lab., Chemistry Department, Faculty of Science, Ain Shams University, Abbassia, Cairo 11566 Egypt

**Keywords:** Benzenesulfonamide, Pyridine moiety, Citrate-AuNPs, Tumour targeting, ^99m^Tc-radiolabeling, Diagnostics, Drug delivery

## Abstract

Sulfonamide derivatives are a significant class of medicinal compounds. Gold nanoparticles (AuNPs) offer precise cancer treatment through targeted delivery, boasting high drug-loading capacity and low toxicity. This study aimed to develop and evaluate 99mTc-labeled benzenesulfonamide derivative-entrapped gold citrate nanoparticles as a tumor-targeting agent. A novel benzenesulfonamide derivative bearing a pyridine moiety was synthesized. Compound 3 (4-((3-cyano-4-(2,4-dichlorophenyl)-6-phenylpyridin-2-yl)amino)-N-(diaminomethylene)benzenesulfonamide) exhibited remarkable anti-cancer activity against MCF-7 cells. The chemical reduction method was employed to create compound 3-citrate-AuNPs. A comprehensive examination of the synthesized nano-platform was conducted, including zeta potential, size analysis, radiochemical yield, and in-vivo biodistribution in tumor-bearing mice. The nano-platform was successfully produced with good stability, optimal particle size (9 nm diameter for AuNPs), and high radiochemical purity for [^99m^Tc]Tc-compound 3 (88.31 ± 2.14%). In-vivo investigations revealed that intravenously administered [99mTc]Tc-compound 3-citrate-AuNPs accumulated in tumors with a high target-to-non-target ratio. The findings validate the efficacy of the novel [^99m^Tc]Tc-compound 3-citrate-AuNPs platform as a tumor-targeting agent.

## Introduction

Cancer remains a formidable threat to human health, affecting millions worldwide^1^. Organic heterocyclic molecules are ubiquitous in pharmaceuticals and medical reagents, offering a broad spectrum of applications. Research has extensively explored the biological effects of heterocyclic compounds on various living organisms. Notably, six-membered heterocyclic derivatives, particularly those containing nitrogen atoms, have garnered significant attention due to their unique properties and diverse applications. Pyridine is a remarkable building component that is required for the synthesis of numerous significant heterocyclic derivatives that include nitrogen. Large pyridine derivatives shown a wide range of biological actions, and many of them are typically employed as very potent medications^2–11^. Since it has been used as an anticancer drug in medicine, benzoenesulfonamide has grown in importance in biology^12,13^. Derivatives of sulfonamides are among the most significant structural classes of medicinal compounds. Sulfonamides have a wide range of biological properties, including anticancer properties^14^. Numerous powerful anticancer medications contain sulfonamide, for example, SLC-0111, a biarylurea-benzenesulfonamide^15^. Because cancer is such a difficult disease to cure, better methods must be found. Benzenesulfonamide derivatives have shown promise in cancer therapy due to their ability to inhibit carbonic anhydrase IX (CA IX), a tumor-associated enzyme. They have also demonstrated antiproliferative activity against various cancer cell lines, including breast, lung, and colon cancer. Additionally, these derivatives have been shown to induce apoptosis in cancer cells and inhibit angiogenesis, making them potential candidates for cancer therapy^16^. Nanotechnology and metal-based medications offer new hope in the fight against cancer. Drugs can be conjugated to nanoparticle surfaces through various methods, including ionic or covalent bonding, structural absorption, or encapsulation into nanoparticle cores^17^. Nanoparticle-based formulations can enhance permeability and retention (EPR) across biological membranes, increasing chemical stability, bioavailability, and absorption. This ensures that the optimal amount of medication reaches the target cancer cells^18–20^. Notably, functionalized gold nanoparticles exhibit remarkable properties, making them highly adaptable for cancer treatment. Gold nanoparticles (AuNPs) are biocompatible^21^ and can be synthesized in various sizes (1–100 nm) and shapes^22^. Their surface can be modified with molecules like antibodies, peptides, or drugs for targeted delivery^23^. AuNPs have a high surface-to-volume ratio, enabling them to carry a large payload of therapeutic molecules^24^. They exhibit unique optical properties, such as surface plasmon resonance (SPR), for imaging, diagnostics, and photothermal therapy^25^. AuNPs are stable, resisting aggregation and degradation^26^, and can be engineered for targeted therapy, reducing side effects^27^. They have shown promise in preclinical studies for targeted chemotherapy and radiotherapy^28^, photothermal therapy^29^, immunotherapy, imaging and diagnostics^30^, and combination therapy. Gold nanoparticles (AuNPs) exhibit remarkable properties^31^, making them an attractive platform for cancer diagnosis and therapy outcomes^32^. With their exceptional drug-loading capacity and low toxicity, AuNPs can enhance diagnostic imaging and cancer treatment outcomes. Furthermore, the precision tuning of AuNPs enables accurate and targeted cancer treatment. This study aims to develop a tracer for identifying and localizing tumors. To achieve this, gold nanoparticles (AuNPs) were synthesized and bifunctionalized with citrate and a novel benzenesulfonamide derivative. The nanoparticles were then in vivo using animal models of tumors.

## Experimental

### Materials and instruments

All of the chemicals and reagents came from sources with a high level of purity. Phosphorous oxychloride (CAS No.: 10025-87-3), phosphorous pentachloride (CAS No.: 10026-13-8), HCl (CAS No.: 7647-01-0), benzene (CAS No.: 71-43-2), sulfaguanidine (CAS No.: 57-67-0), dioxane (CAS No.: 123-91-1), ethanol (CAS No.: 64-17-5), NaBH4 (CAS No.: 16940-66-2), Acetone (CAS No.: 67-64-1), NH4OH (CAS No.: 1336-21-6), chloroauric acid (CAS No.: 16961-25-4), trisodium citrate (CAS No.: 6132-04-3) were purchased from Merck Darmstadt, Germany. Nuclear-magnetic-resonance (13C-NMR, Jeol JNM-Ex-67.8 MHz FT NMR, Jeol Ltd), and (1H-NMR, Jeol EX-270 MHz FT, Jeol Ltd). Paper chromatography (PC) (Whatman International Ltd, Maidstone, Kent, UK). A NaI (Tl) γ-ray scintillation counter (ScalerRatemeter SR7, Nuclear Enterprises, Edinburgh, UK) for γ-ray radioactivity measurement. Albino mice, each of 20–25 g, were used for the in-vivo study. The IR spectrum was executed on a Perkin-Elmer 293 spectrophotometer. Mass spectrometric (EI, 70 eV) analysis. Microanalytical analyses (CHN) were executed on a Perkin-Elmer analyzer (CHN-2400). JEOL-JEM 1010 TEM and Malvern-Zetasizer (Z.S. 90) were used for nanoparticles characterization. ScalerRatemeter SR7 model (NaI(T.I.) gamma counter) was used for radioactivity determination. Albino mice were used for biodistribution study. The Radioactive Isotopes Production Facility (RPF) in Egypt was the source of technetium-99m.

### Synthesis

#### Synthesis of 2-chloro-4-(2,4-dichlorophenyl)-6-phenylnicotinonitrile (2)

Phosphorous oxychloride (1.53 mL, 0.01 mol), phosphorous pentachloride (2.08 g, 0.01 mol), and compound 1 (4-(2,4-dichlorophenyl)-2-oxo-6-phenyl-1,2-dihydropyridine-3-carbonitrile) (3.4 g, 0.01 mol) were refluxed over a water bath for two hours. The liquid was placed into diluted ice HCl after cooling. The resulting solid was filtered, washed with water, and then recrystallized from benzene to yield compound 2.

Beige powder, IR (KBr, υ, cm^−1^): 1592 (C=C), 1648 (C=N), 2226 (CN). ^13^C-NMR (DMSO-d_6_) δ: 115.5, 120.5, 121.0, 127.7, 128.0, 129.1, 129.2, 129.5, 131.7, 132.0, 132.4, 133.2, 134.2, 135.1, 135.9, 151.7, 153.3, and 159.5. ^1^H-NMR (DMSO-d_6_) δ: 6.76–8.22 (m, 9H, Ar-H). Anal. found for C_18_H_9_ N_2_Cl_3_: C, 59.96; H, 2.54; N, 7.67; Cl, 29.83. MS: m/z 359 (M^+^) (22.53%). m.p. = 120–122 °C.

#### Synthesis of 4-((3-cyano-4-(2,4-dichlorophenyl)-6-phenylpyridin-2-yl)amino)-N-(diaminomethylene)benzenesulfonamide (3)

A mixture of compound 2 (0.01 mol, 3.59 g) and sulfaguanidine (0.01 mol, 2.14 g) in dioxane was refluxed in a water bath for 7 h. After cooling, the resulting material was filtered, collected, washed with ethanol, and then recrystallized from a suitable solvent to yield compound 3.

Buff crystals from ethanol, m.p. = 222–224 °C. IR (KBr, υ, cm^−1^): 3429, 3333, 3258 (NH2), 3062 (NH), 2207 (CN), 1591 (C=N). 1H-NMR (DMSO-d6) δ: 6.66 (s, 4H, 2NH2, D2O exchangeable), 7.15–8.18 (m, 13H, Ar-H), 8.55 (s, 1H, NH, D2O exchangeable). 13C-NMR (DMSO-d6) δ: 88.7, 110.2, 113.6, 114.0, 115.3, 120.5, 121.4, 125.6, 1271, 127.8, 128.2, 129.0, 129.7, 130.5, 131.2, 131.9, 132.1, 132.4, 132.8, 133.2, 135.9, 137.2, 151.8, 154.4, and 158.0. MS: m/z 537 (M+) (14.72%). Anal. Calcd. for C25H18O2N6SCl2: C, 55.87; H, 3.35; N, 15.64; S, 5.96; Cl, 13.22; Found: C, 55.92; H, 3.28; N, 15.68; S, 5.93; Cl, 13.33.

### Preparation of [^99m^Tc]Tc-compound 3

Using the direct radiolabeling method, Compound 3 was radiolabeled with the gamma-emitting, short-half-life ^99m^Tc. To maximize radiochemical purity (RCP) and efficiency, parameters influencing the radiolabeling process were thoroughly investigated. The following parameters were examined: substrate amounts (100–800 µg), NaBH_4_ concentrations (2.5–20 mg), and pH ranges (6–10) over a 6-h period. Paper chromatography (PC) was employed to monitor the RCP of the [^99m^Tc]Tc-compound 3 complex using two distinct mobile phases. Acetone was used to detect free pertechnetate, which migrated to Rf = 1, while the other component remained at Rf = 0. A second mobile phase, comprising NH_4_OH, C_2_H_5_OH, and H_2_O (1:2:5), was used to determine the reduced hydrolyzed (R-H) 99mTc-colloid, which remained at Rf = 0, whereas labeled and free compounds migrated to Rf = 1^33,34^.

### Synthesis of [^99m^Tc]Tc-compound 3-citrate-AuNPs and characterization techniques

**Scheme 1:**
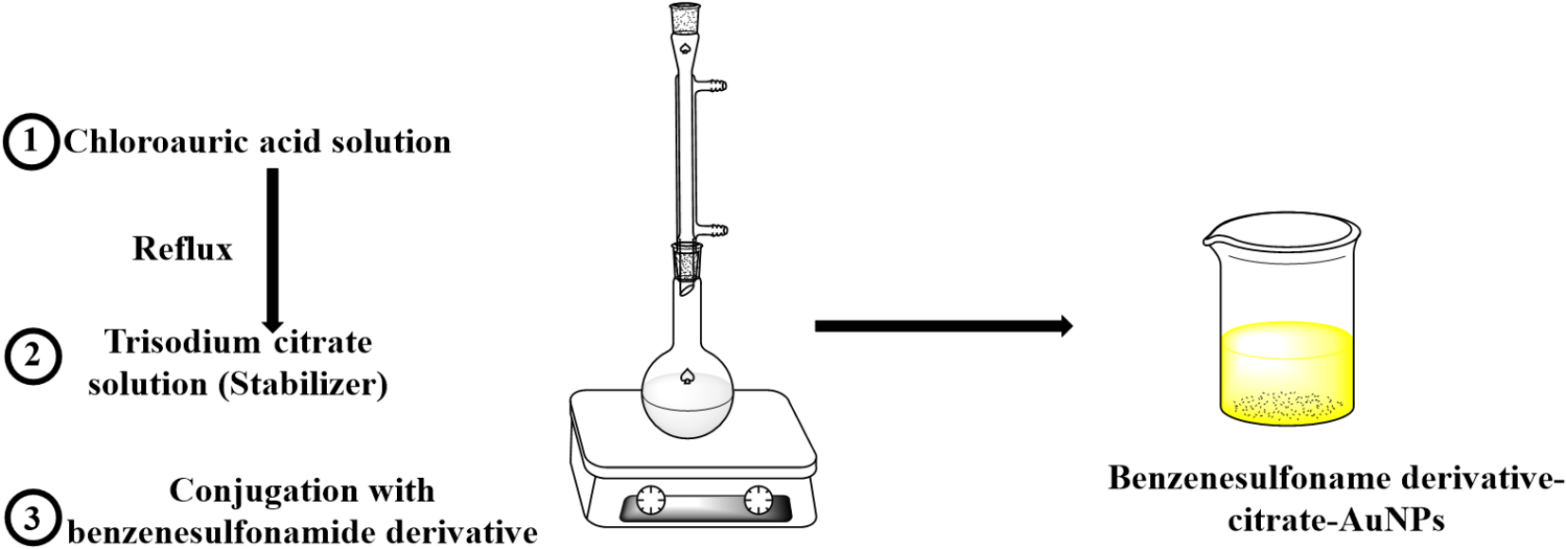
Schematic representation of the grafting process for gold nanoparticles.

A 0.10 mM chloroauric acid solution (10 mL) was heated under reflux to its boiling point, then 0.7 mL of 38.80 mM trisodium citrate solution was added dropwise while being vigorously stirred mechanically for 30 min. After centrifugation at 13,500 rpm for 20 min, the resulting citrate-stabilized gold nanoparticles (citrate-AuNPs) were re-suspended in double-distilled water for further study^35^ (Scheme 1). Subsequently, [^99m^Tc]Tc-compound 3 solution was added dropwise to the colloidal AuNPs under ultrasonication in a 1:1 ratio to form [^99m^Tc]Tc-compound 3-citrate-AuNPs. The loading efficiency was then calculated^36^.

### Stability study

#### Stability at room temperature

Multiple vials of [^99m^Tc]Tc-compound 3-citrate-AuNPs were prepared and stored at room temperature (20–25 °C) in a controlled environment to assess their stability under these conditions.

#### Stability in human serum

The stability of the complex in human serum was investigated in vitro by mixing 0.2 mL of the complex with 1.8 mL of human serum at 37 °C, followed by analysis at multiple time points (1–24 h) to determine serum stability.

### In-vitro anticancer evaluation

Three human tumour cell lines, namely MCF-7 (mammary gland breast cancer), were obtained from the American Type Culture Collection (ATCC) via the Holding Company for Biological Products and Vaccines (VACSERA), Cairo, Egypt. MCF-7 cells provide a suitable model for evaluating the anticancer properties of benzenesulfonamide derivatives, especially in the context of hormone-dependent breast cancer. MCF-7 cells were selected for this study due to their relevance to human breast cancer, well-established characterization, and hormone receptor-positive status. Additionally, their sensitivity to anticancer agents and ease of culture make them a suitable model for assessing the efficacy of new compounds^37,38^. The tumor cell lines were seeded in Corning 96-well tissue culture plates at a density of 5 × 10^4^ cells/well and incubated for 24 h. Subsequently, the tested substances were added to the wells in triplicate. Cell viability was assessed using the MTT assay.

### Biodistribution study of [^99m^Tc]Tc-compound 3-citrate-AuNPs

The research ethics committee at Ain Sams University in Egypt approved all experimental protocols and animal operations (code: ASU-SCI/CHEM/2024/8/1). All experiments were conducted in accordance with relevant guidelines and regulations, complying with the International Guiding Principles for Biomedical Research Involving Animals^39^. This study adheres to the ARRIVE guidelines for reporting in vivo experiments^40^. Tumour induction was performed using the Ehrlich ascites carcinoma cell line (EAC), which was derived from a mouse mammary cancer^41^. Swiss Albino mice, aged three months and weighing 20–25 g were used. The mice used in the experiment were provided and housed by the animal house associated with the Isotopes Production and Radioactive Sources Division, hot labs centre for the duration of the experiment. Following isolation from a female Swiss Albino donor mouse, the Ehrlich ascites carcinoma (EAC) cells were diluted with sterile saline. Each mouse (weighing 20–25 g) received a 100 µL intramuscular injection of EAC solution into the left thigh^42^. Subsequently, a 50 µL solution containing the [^99m^Tc]Tc-compound 3-citrate-AuNPs complex was administered via tail vein injection. At predetermined time points post-injection (15, 30, 60, 120, 240 min, and 24 h) 3 mice at each point, the mice were euthanized, and relevant organs were harvested, weighed, and counted. Results were expressed as percentage injected dose per gram of organ (% ID/g).

## Results and discussion

### Chemistry

**Scheme 2:**
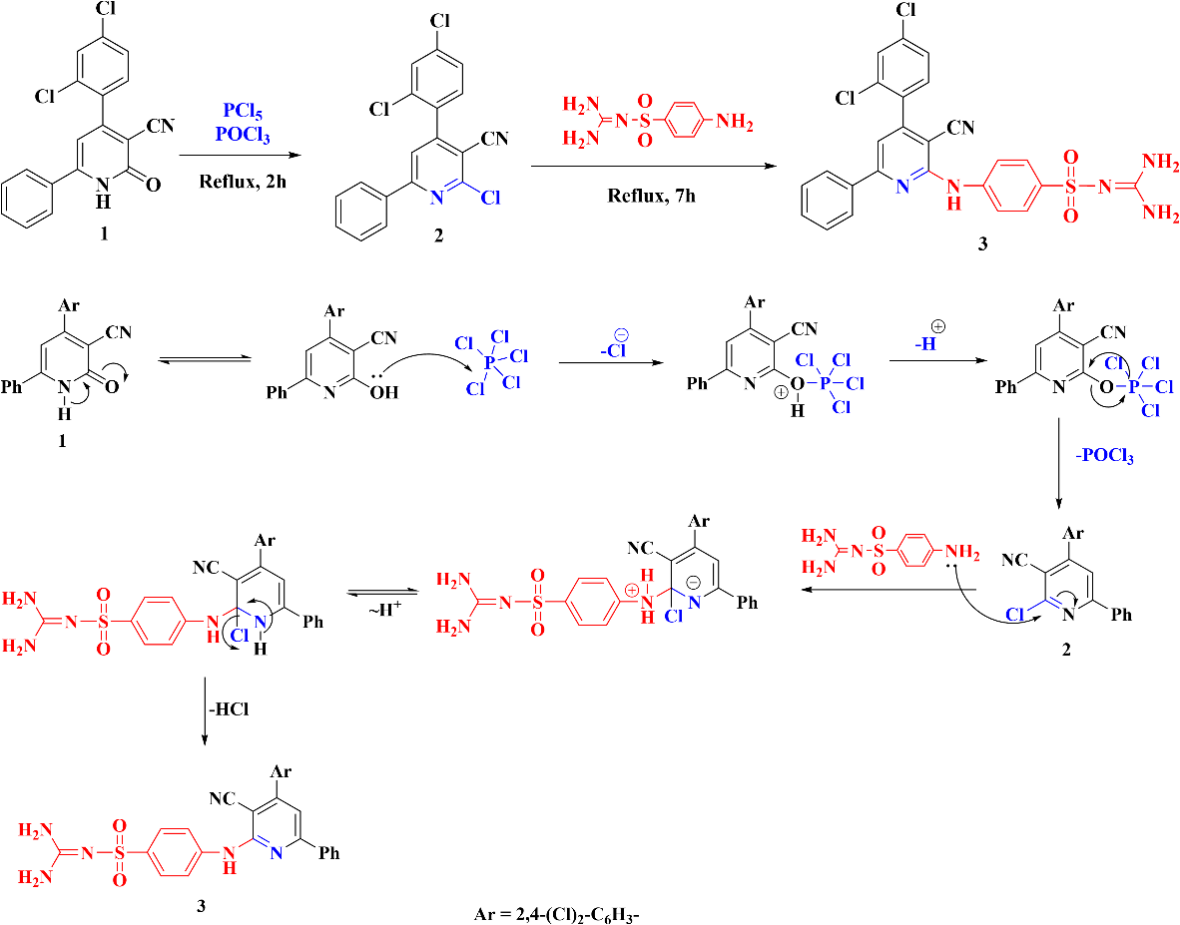
Synthesis of 4-((3-cyano-4-(2,4-dichlorophenyl)-6-phenylpyridin-2-yl)amino)-N-(diaminomethylene)benzenesulfonamide.

The synthetic route outlined in Scheme 2 was employed for the preparation of the novel derivative, following established protocols^43–45^, 4-(2,4-dichlorophenyl)-2-oxo-6-phenyl-1,2-dihydropyridine-3-carbonitrile 1 was produced as the starting material. The desired pyridine derivative 2 was synthesized by reacting compound 1 with phosphorus pentachloride in the presence of phosphorus oxychloride.

Following the reaction of compound 2 with sulfaguanidine, a nitrogen nucleophile, the resulting product 3 was formed via a nucleophilic aromatic substitution mechanism. Specifically, the amino group of sulfaguanidine attacked the C2 position of the pyridine ring, accompanied by the elimination of one HCl molecule (Scheme 2).

### Radiolabeling of compound 3 with technetium-99m

**Fig. 1:**
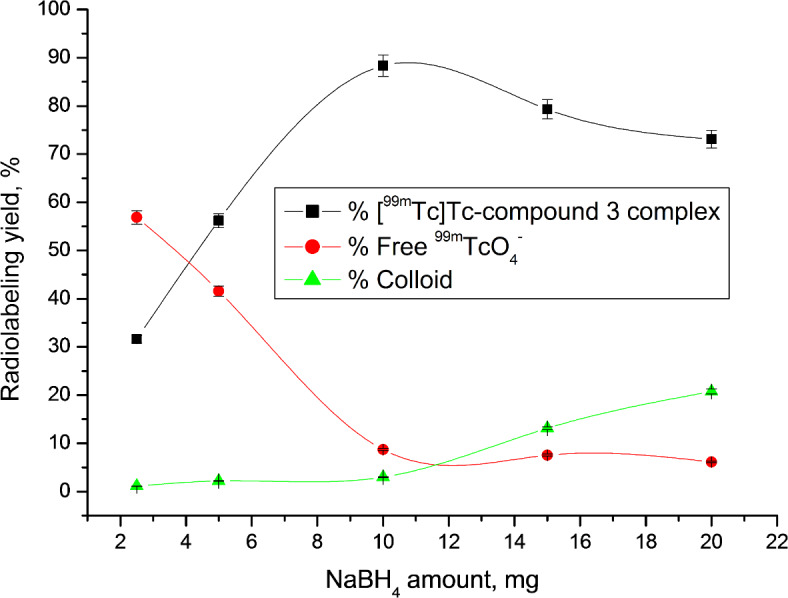
Radiolabeling yield of [^99m^Tc]Tc-compound 3 complex as a function of NaBH_4_ amount.

**Fig. 2:**
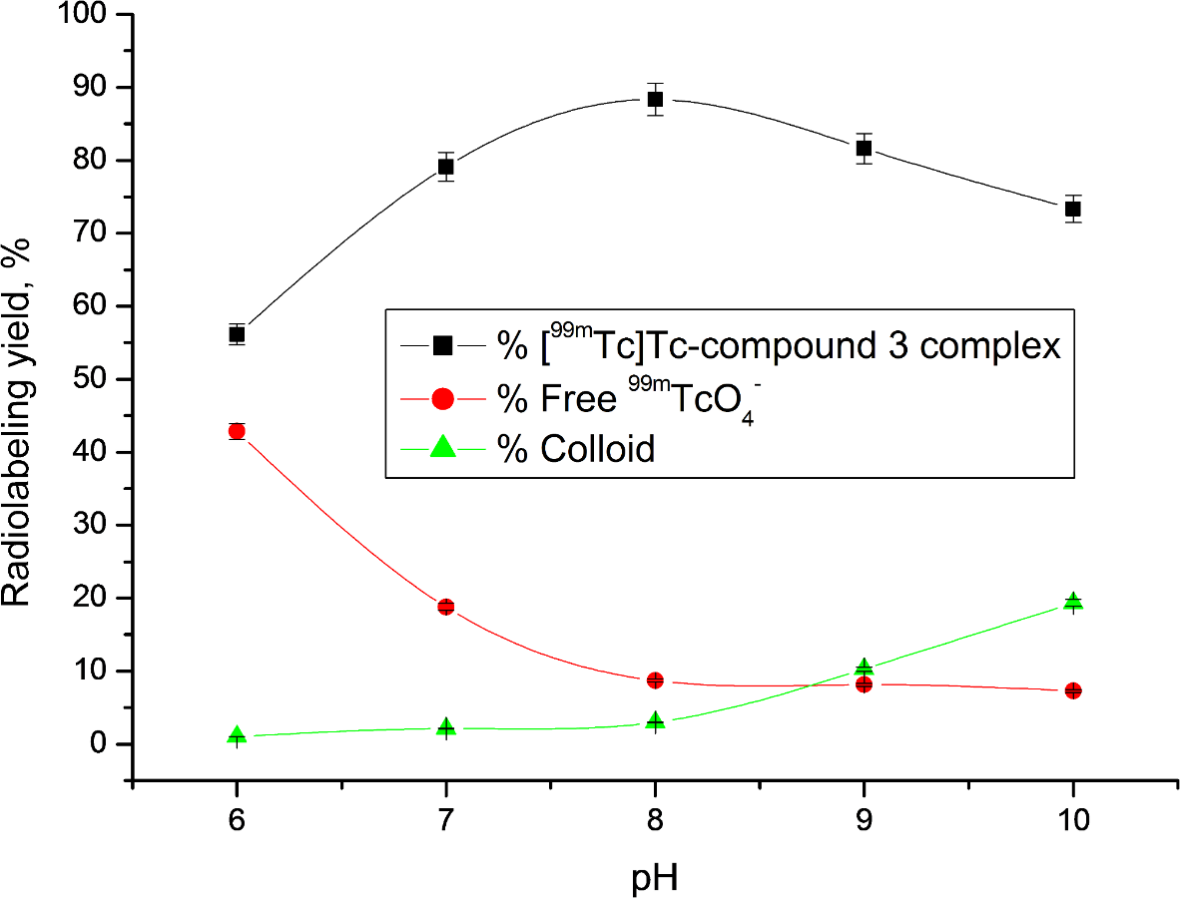
Radiolabeling yield of [^99m^Tc]Tc-compound 3 complex as a function of pH.

**Fig. 3:**
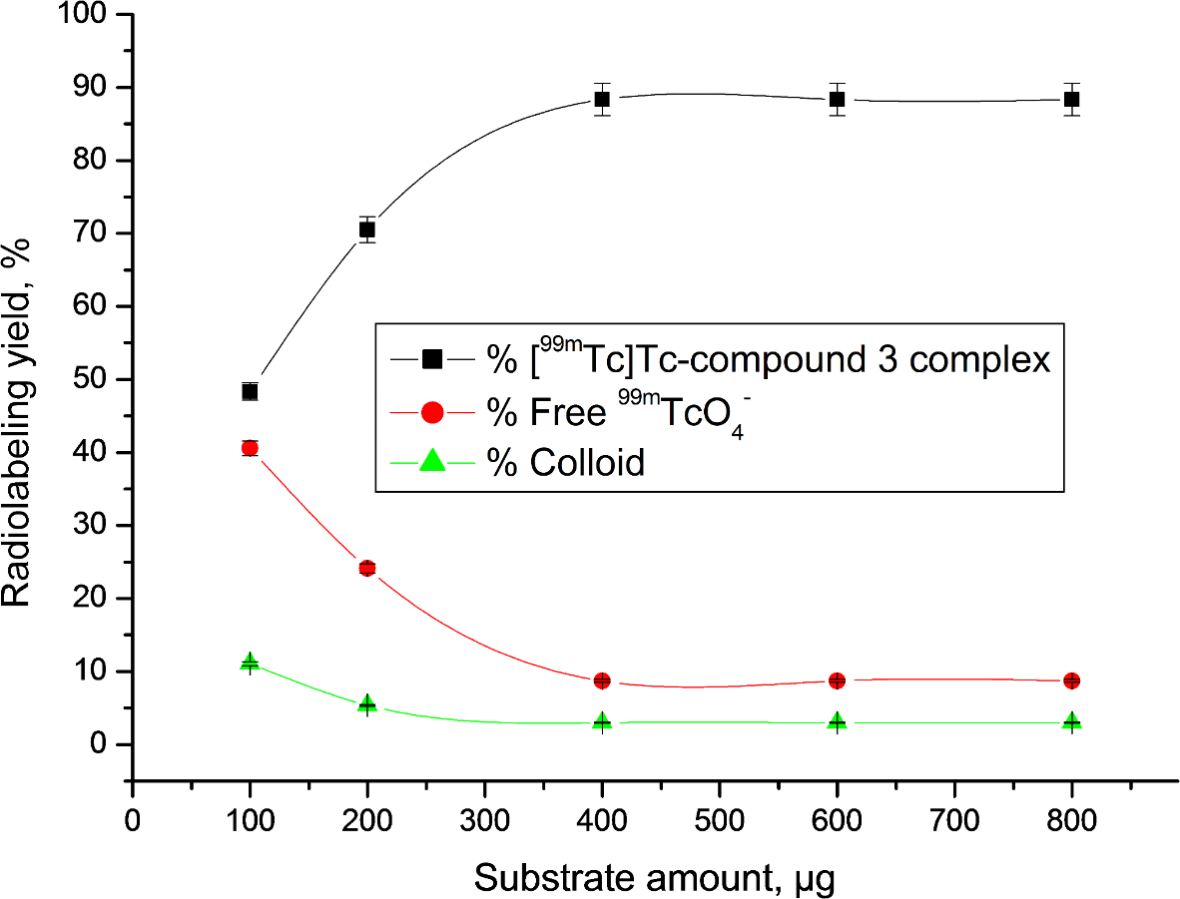
Radiolabeling yield of [^99m^Tc]Tc-compound 3 complex as a function of substrate amount.

**Fig. 4:**
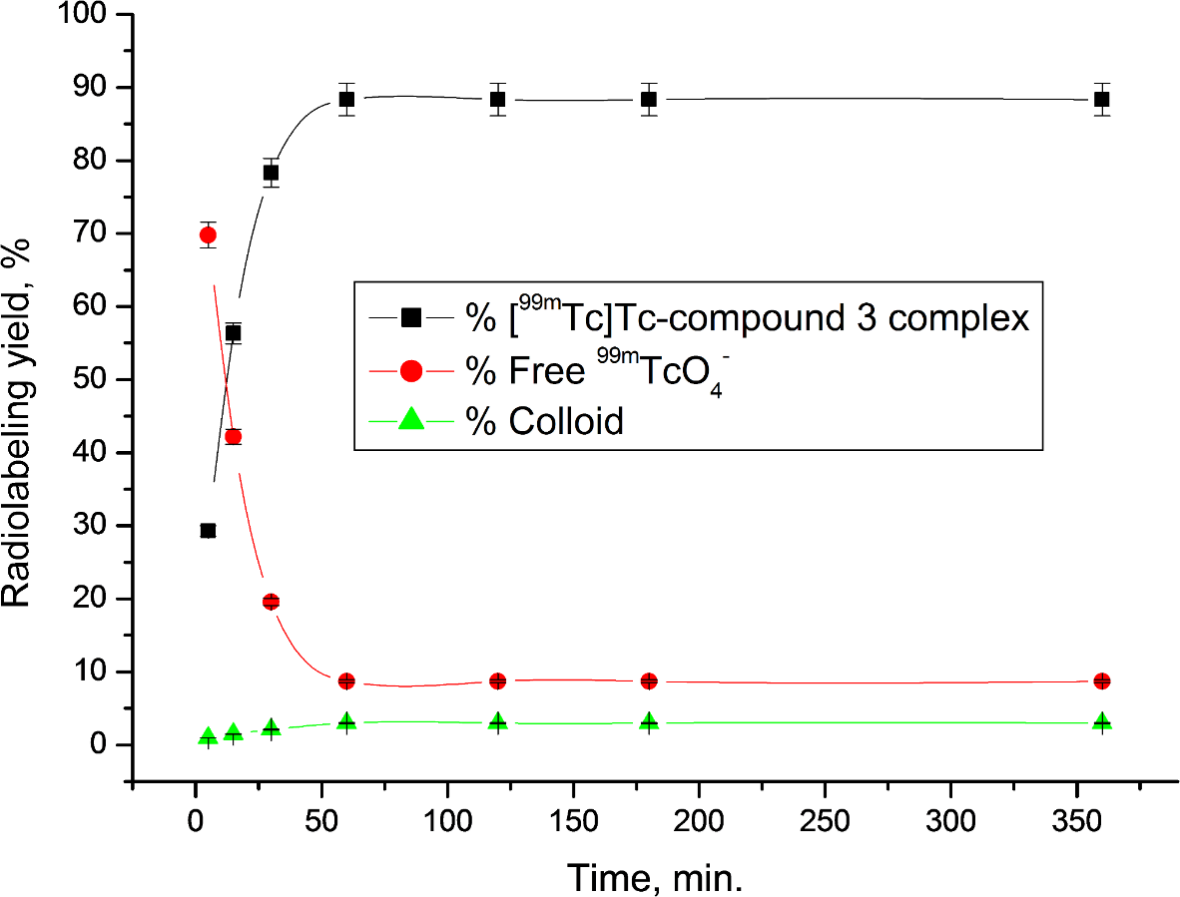
Radiolabeling yield of [^99m^Tc]Tc-compound 3 complex as a function of time.

A paper chromatography approach was employed to evaluate the radiochemical purity (RCP) of the [^99m^Tc]Tc-compound 3 complex^46^. Optimization studies revealed that the ideal substrate amount was 400 µg, which, after a 1-hour reaction at room temperature and using 10 mg NaBH_4_ at pH 8, yielded a complex with 99mTc having the highest RCP of 88.31%. The reduction of ^99m^Tc from its heptavalent oxidation state to a suitable state was achieved using NaBH_4_ as a reducing agent, enabling complexation with the legend^47^. However, using higher amounts of NaBH4 (> 10 mg) resulted in decreased RCP due to increased colloid formation, while lower amounts (< 10 mg) were insufficient to reduce all TcO4- in the solution, also yielding lower RCP values (Fig. 1). Another critical factor in radiosynthesis research is pH. At pH 10, the radiochemical purity (RCP) decreased to 73.33% due to colloid formation (19.36%). In contrast, at pH values lower than 8, colloid formation decreased, while free 99mTcO4- increased (Fig. 2). The quantity of substrate also significantly impacted RCP ratios. Lower substrate content resulted in lower RCP, as insufficient chelating agent was available to form a complex with reduced 99mTc. However, substrate amounts exceeding the optimal value had no discernible effect on RCP (Fig. 3). The optimal reaction time was found to be 1 h, and the complex remained stable after 360 min (Fig. 4).

### Synthesis of [^99m^Tc]Tc-compound 3-citrate-AuNPs

**Fig. 5:**
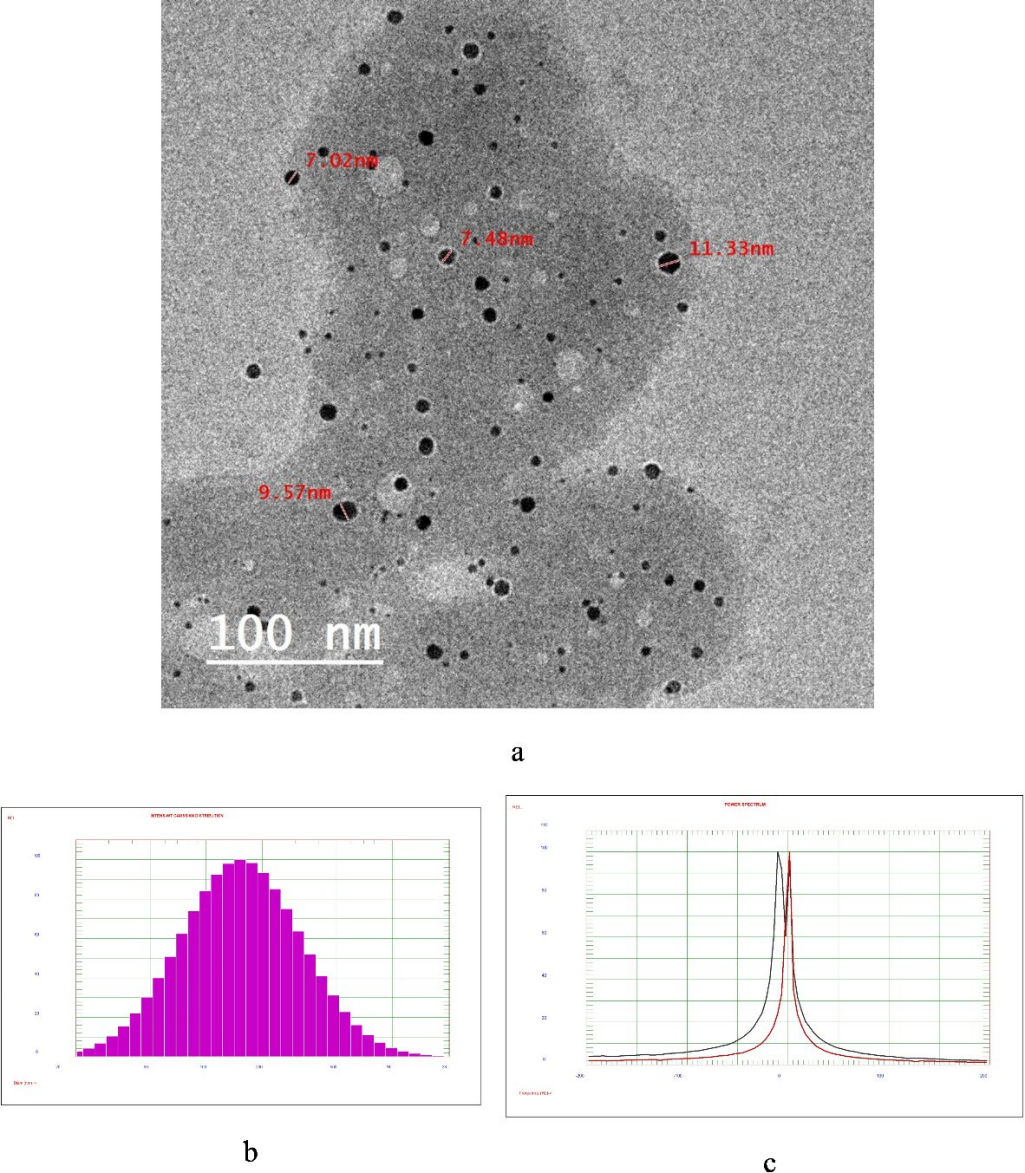
(**a**) citrate-AuNPs TEM image, (**b**) citrate-AuNPs DLS, (**c**) citrate-AuNPs Zeta potential.

Citrate-stabilized gold nanoparticles (citrate-AuNPs) were synthesized using a previously established procedure^35^. The reducing and stabilizing action of sodium citrate prevented nanoparticle aggregation by converting Au3+ into elemental gold (Au0). Transmission electron microscopy (TEM) revealed that the resulting nanoparticles had an average size of approximately 9 nm, with a hydrodynamic diameter of 203.1 nm. The measured zeta potential, an indicator of suspension stability, was − 11.84 mV (Fig. 5). The zeta potential measures the electrostatic potential generated by ion accumulation at particle or cell surfaces in a fluid. It indicates stability and surface properties in colloidal systems. A zeta potential of − 11.84 mV signifies: A net negative surface charge due to the negative zeta potential and Moderate stability^48–50^. The loading capacity of Compound 3 was determined by measuring the radioactivity in the precipitate ([^99m^Tc]Tc-compound 3-citrate-AuNPs) and supernatant ([^99m^Tc]Tc-compound 3). The loading efficiency was calculated by dividing the radioactivity of the precipitate by the total radioactivity (precipitate plus supernatant). A high loading capacity of 83.2% was achieved after 1 h.

### Stability study at room temperature and in human serum

The complex [^99m^Tc]Tc-compound 3-citrate-AuNPs was accepted for this study according to the following criteria for both room temperature stability and stability in human serum: the radiochemical purity should remain ≥ 90% throughout the study, the radioactivity concentration should remain within ± 10% of the initial value, and the pH stability should be maintained within a narrow range of ± 0.5 pH units throughout the study^51^.

### In-vitro anticancer evaluation

Breast cancer being one of the most prevalent forms of cancer in humans, we selected the MCF-7 human breast cancer cell line as a model for our study. The in vitro cytotoxicity of the synthesized enaminonitrile pyridine derivatives against MCF-7 cells was evaluated. Cisplatin was used as a reference standard to compare the cytotoxic potency. The results showed that compound 3 exhibited cytotoxicity against MCF-7 cells with an IC50 value of 47.17 ± 4.12. Notably, the IC50 value of compound 3-citrate-AuNPs was significantly lower (8.41 ± 0.36), indicating enhanced cytotoxicity.

### Biodistribution study of [^99m^Tc]Tc-compound 3-citrate-AuNPs

**Fig. 6:**
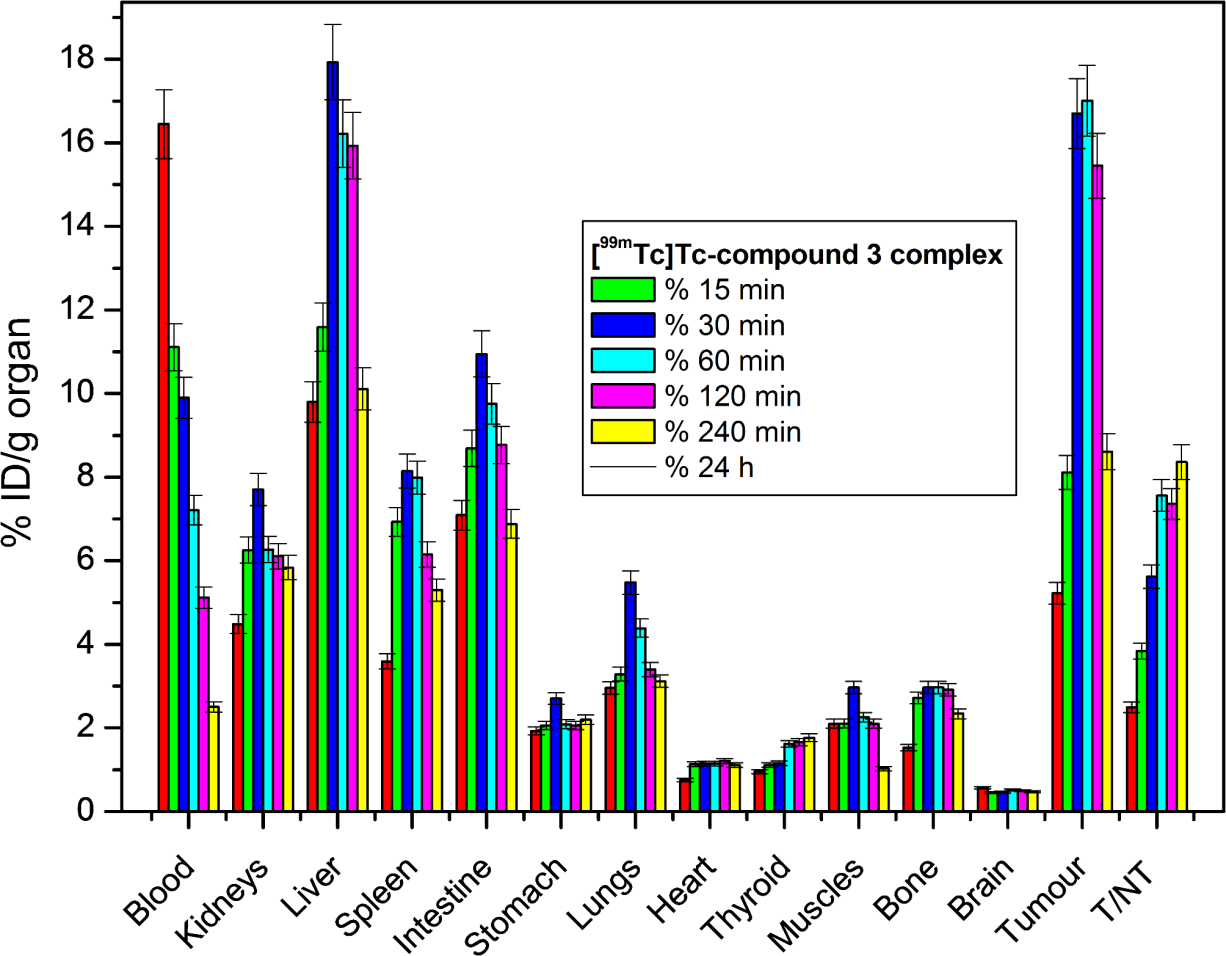
Biological distribution of [^99m^Tc]Tc-compound 3 complex in solid tumour bearing Albino mice.

The biodistribution of the [^99m^Tc]Tc-compound 3-citrate-AuNPs complex was evaluated in solid tumor-bearing mice (Fig. 6). The results showed that the complex accumulated predominantly in the tumor site, with a maximum uptake of 17.01% ID/g at 2 h. The complex’s clearance pathway was characterized by high liver uptake (17.93% ID/g at 60 min) and kidney accumulation (7.70% ID/g at 60 min). The liver’s unique characteristics, including high blood flow and phagocytic activity, make it a primary site for nanoparticle accumulation. Additionally, the physical and chemical properties of nanoparticles, such as size, charge, and surface chemistry, influence their interaction with liver cells and subsequent retention. As a result, nanoparticles are often cleared from the bloodstream and sequestered in the liver, where they may be metabolized or eliminated^52,53^. To minimize liver accumulation of nanoparticles, researchers employ various strategies, including modifying their surface chemistry, optimizing their size and shape, and utilizing targeted delivery methods. Additionally, adjusting the dosage and administration route can also help reduce liver uptake. By leveraging these approaches, nanoparticles can be engineered to maximize their therapeutic potential while minimizing liver accumulation^54–57^. The complex’s high lipophilicity and large particle size likely contributed to its primary excretion via the hepatobiliary pathway. The target-to-non-target (T/NT) (The Tumor-to-Normal tissue ratio measures the selective uptake and evaluates its efficacy and specificity in cancer diagnosis and therapy) was assessed by comparing tumor muscle with normal muscle (Fig. 6). A high T/NT ratio of 7.56 was observed at 120 min post-injection, indicating potential for solid tumor targeting. The [^99m^Tc]Tc-compound 3-citrate-AuNPs exhibits superior accumulation compared to several developed agents, as evident from its higher T/NT values, surpassing those of ^99m^TcN-MAGAMCPP^58^, ^99m^Tc-sunitinib^59^, ^99m^Tc-cyclohexane dioxime derivative^34^, pyrazolone derivative^60^, ^99m^Tc-amitrole^42^ and gold(III) complex on nanoporous MCM-41^61^.

## Conclusion

This study aimed to synthesize a novel pyridine derivative (compound 3) as a potent anticancer agent. The [^99m^Tc]Tc-compound 3 complex, with a radiochemical purity (RCP) of 88.31%, was successfully loaded onto citrate-stabilized gold nanoparticles (citrate-AuNPs) with a core size of 9 nm, demonstrating high loading efficiency and stability. In vivo studies revealed that intravenous administration of the [^99m^Tc]Tc-compound 3-citrate-AuNPs complex resulted in significant accumulation at the tumor site with a high target-to-non-target ratio. These promising findings underscore the potential of the [^99m^Tc]Tc-compound 3-citrate-AuNPs complex as a tumor-targeting agent.

## Data Availability

The data used to support the findings of this study are available from the corresponding author upon request.
